# *In Vivo* Tracking of Transplanted Mononuclear Cells Using Manganese-Enhanced Magnetic Resonance Imaging (MEMRI)

**DOI:** 10.1371/journal.pone.0025487

**Published:** 2011-10-07

**Authors:** Kenichi Odaka, Ichio Aoki, Junji Moriya, Kaoru Tateno, Hiroyuki Tadokoro, Jeff Kershaw, Tohru Minamino, Toshiaki Irie, Toshimitsu Fukumura, Issei Komuro, Tsuneo Saga

**Affiliations:** 1 Molecular Imaging Center, National Institute of Radiological Sciences, Chiba, Japan; 2 Department of Cardiovascular Science and Medicine, Chiba University Graduate School of Medicine, Chiba, Japan; 3 Department of Bio-Medical Engineering, Tokai University, Shizuoka, Japan; St. Georges University of London, United Kingdom

## Abstract

**Background:**

Transplantation of mononuclear cells (MNCs) has previously been tested as a method to induce therapeutic angiogenesis to treat limb ischemia in clinical trials. Non-invasive high resolution imaging is required to track the cells and evaluate clinical relevance after cell transplantation. The hypothesis that MRI can provide *in vivo* detection and long-term observation of MNCs labeled with manganese contrast-agent was investigated in ischemic rat legs.

**Methods and Findings:**

The Mn-labeled MNCs were evaluated using 7-tesla high-field magnetic resonance imaging (MRI). Intramuscular transplanted Mn-labeled MNCs were visualized with MRI for at least 7 and up to 21 days after transplantation in the ischemic leg. The distribution of Mn-labeled MNCs was similar to that of ^111^In-labeled MNCs measured with single-photon emission computed tomography (SPECT) and DiI-dyed MNCs with fluorescence microscopy. In addition, at 1–2 days after transplantation the volume of the site injected with intact Mn-labeled MNCs was significantly larger than that injected with dead MNCs, although the dead Mn-labeled MNCs were also found for approximately 2 weeks in the ischemic legs. The area covered by CD31-positive cells (as a marker of capillary endothelial cells) in the intact Mn-MNCs implanted site at 43 days was significantly larger than that at a site implanted with dead Mn-MNCs.

**Conclusions:**

The present Mn-enhanced MRI method enabled visualization of the transplanted area with a 150–175 µm in-plane spatial resolution and allowed the migration of labeled-MNCs to be observed for long periods in the same subject. After further optimization, MRI-based Mn-enhanced cell-tracking could be a useful technique for evaluation of cell therapy both in research and clinical applications.

## Introduction

Cell therapy to treat cardiovascular disease has come of age. For instance, bone marrow-derived mononuclear cells (MNCs) have been used for therapeutic neovascularization not only in animal models[1], [2], [3], [4], but also in the clinical setting[5], [6]. Peripheral blood MNCs have also been used to induce therapeutic neovascularization for critical limb ischemia[7], [8] and myocardial infarction[9], [10]. However, methods that can be used to reliably evaluate therapeutic effects and migration of transplanted MNCs are not well established. *In vivo* monitoring of the healing process after cell transplantation, particularly the fate of transplanted cells, is required for high-precision optimization in order to improve the efficacy of such cell therapies. In addition, non-invasive imaging of transplanted MNCs may contribute to understanding the mechanism underlying therapeutic effects such as angiogenesis[11].

There is a rapidly growing interest in tracking cell movements both in animals and humans[12], [13]. Tracking MNCs with magnetic resonance imaging (MRI) has been used in living tissues such as skeletal muscle, heart and brain to visualize both the regenerative therapeutic effect and the location of migrated cells with a high spatial resolution[14], [15], [16]. Iron oxide nanoparticles can enhance cell visualization because the susceptibility difference significantly alters T_2_*, especially in high field MRI[17]. Dextran-coated iron oxide nanoparticles have been found to have a wide clinical application for detection of hepatic tumors[18]. On the other hand, iron oxide particles still have several shortcomings as cell-labeling agents. First, the iron oxide particles stay inside cells for long periods[19], become engulfed by cardiac macrophages[20], and do not indicate cell viability. Second, iron oxide particles often provide negative contrast that is difficult to distinguish from ‘dark regions’ in the body, such as air cavities, veins and other regions where there is intrinsic iron deposition after injury. Third, iron oxide particles need specialized materials and methods for cell labeling such as vectors, [21] transfection reagents[22] and electroporation[23].

Manganese (Mn) is known to be a toxic substance that causes ‘manganism’ through chronic exposure in environments such as mines[24]. The divalent manganese ion (Mn^2+^) is also known to be essential for living organisms. For this reason, there has been a recent renewed interest in Mn^2+^ as a potentially useful positive contrast agent for T_1_ weighted MRI. The kinetics of Mn^2+^ in the cell mimics the kinetics of calcium ions (Ca^2+^) in many biological systems[25], [26], as Mn^2+^ is known to enter cells through ligand- or voltage-gated Ca^2+^ channels[27]. Recently, Mn^2+^ agents have found application with manganese-enhanced MRI (MEMRI) for visualization of many biological features, including neuronal pathways[28] and neuro/cytoarchitecture[29], [30].

Previous work has used the fact that Mn^2+^ can enter cells via voltage gated Ca^2+^ channels during stimulation in order to enhance excitable cells in the brain[31], [32], and to monitor changes in inotropic status[33] or ischemic disorders[34] in the heart. The fact that Mn^2+^ can enter properly functioning cells has led to work aimed at developing Mn^2+^ or Mn-dipyridoxyl-diphosphate (Mn-DPDP) as cell viability indicators for cardiac applications[35]. Mn^2+^ has also been applied to immunocyte labeling *in vitro* and the toxicity, immunoreactivity, and relaxivity for MRI have been tested[36]. All of this work has indicated that Mn^2+^ is a useful contrast agent for cellular imaging.

On the basis of prior *in vitro* work using manganese chloride (MnCl_2_) to label cells for MRI[36], a MEMRI-based technique has been developed for tracing transplanted-peripheral blood-derived MNCs *in vivo*. In the present report, the possibility that MNCs can be labeled with MnCl_2_ contrast agent to a level sufficient to allow *in vivo* detection and tracking by T_1_-weighted MRI was tested in the ischemic legs of rats. Kinetics of the intramuscularly transplanted MNCs were visualized and evaluated using MEMRI, indium-111 (^111^In) oxine single-photon emission computed tomography (SPECT) and fluorescence microscopy. In order to evaluate the therapeutic effects of the MNCs, blood flow was measured in the ischemic leg muscle using laser Doppler perfusion imaging, MR angiography and immunohistochemical analysis for CD31 (platelet endothelial cell adhesion molecule).

## Materials and Methods

All study was performed under a protocol approved by the Animal Welfare and Use Committee of the National Institute of Radiological Sciences (Japan, 07-1067-5) and based on the Helsinki Declaration. Male Wistar rats (114–284 g, 6 to 16 weeks, n = 18, donors  =  81, SLC, Japan) were used. For visualization of transplanted MNCs, 2 experiments (short-term and long-term observations) were performed using a hindlimb ischemia model in the rat. For the short-term observation experiment, 2 groups of rats were used. The control group (n = 3) was administered saline while the other group was given Mn-labeled MNCs (n = 3, MNC donors  =  9). For the long-term observation experiment, 3 groups of rats were administered different substances: a Mn-labeled MNC group with intact MNCs transplanted into the left hindlimb and dead MNCs into the right hindlimb (n = 6, MNC donors  =  36), an intact ^111^In-labeled MNCs transplanted group for SPECT imaging (n = 1, MNC donors  =  6), and an intact 1,1′-dioctadecyl-3,3,3′,3′-tetramethylindocarbocyanine (DiI)-labeled MNCs transplanted group for fluorescent microscopic imaging (0, 2, 14, 21, and 28 days, n = 1 for each, MNC donors  =  30).

### Mn and DiI labeling for peripheral blood MNCs

Donor rats were euthanized with a lethal dose of a pentobarbital (150 mg/kg, Dainippon-Sumitomo Pharmaceutical, Osaka, Japan), after which heparinized whole blood was harvested. MNCs were subsequently separated using Histopaque 1083 (Sigma-Aldrich, St. Louis, MO, USA)[7]. Osmotic pressure controlled MnCl_2_·4H_2_O (Sigma-Aldrich) was dissolved in PBS with 5% serum and prepared at 0.25 mM MnCl_2_ for the hindlimb ischemia model[36]. MNCs were mixed with the MnCl_2_ solutions and incubated for 60 min at 37°C. For the long-term experiment, a subgroup of cells was marked with a fluorescent (DiI) dye (Life Technologies Japan, Tokyo, Japan) at a concentration of 2 µg/ml[37]. DiI was added to PBS with 5% serum and MnCl_2_ 5 min before the end of incubation. After incubation, the MnCl_2_ solution was removed carefully by washing twice using PBS with 5% serum. Half of the Mn-labeled MNCs were heated in 90°C water for the dead Mn-labeled MNCs group. Cell viability was determined by staining with trypan-blue and manually counting the living and dead cells using an optical microscope.

### Animal models

Rats were initially anesthetized with 4.0% isoflurane (Abbott Japan, Tokyo, Japan), and then kept anesthetized with 2.0% isoflurane mixed with a 1∶7 O_2_/room-air gas mixture using a facemask. Rectal temperature was maintained at approximately 37.5°C by an automatic heating system. The proximal part of the femoral artery and the distal portion of the saphenous artery were ligated based on the procedure described in a previous report[38]. The intact Mn-labeled MNCs were injected into the left ischemic hindlimb (7.86±5.40×10^6^ cells in 100 µL) 6 h after the ischemia. Dead Mn-labeled MNCs (7.86±5.40×10^6^ cells in 100 µL) or saline (100 µL) were injected into the right ischemic hindlimb. Paled skin and decreased skin temperature were observed bilaterally in the hind legs after arterial occlusion. The ability of the rats to feed and use their legs was monitored every day up until the experiments were performed. Abnormal gait was typically observed for 1 week after treatment in all hindlimb ischemic models. No symptoms of infection or amputation were seen in the rats throughout the study.

### MRI measurements

#### *In vitro* MRI measurements

Mn-labeled MNCs (0, 0.1, 0.25, and 0.5 mM, 60 min incubation, washed twice with phosphate-buffered saline (PBS)) were put into 0.2-mL tubes with references consisting of saline, 0.1, 0.25, 0.5 mM MnCl_2_ solution, and the Mn solution remaining after cell-labeling (supernatant fluid). The MNCs were imaged after centrifuging into pellet form. The MRI acquisitions were performed 1 h after the cell preparation with a 7.0-T, 40-cm bore magnet (JASTEC-Kobelco, Tokyo, Japan) interfaced to a Bruker console (Bruker Biospin, Ettlingen, Germany). A 75-mm-diameter birdcage transmitting/receiving coil (Bruker-Biospin) was used for sample measurement. The polymerase chain reaction (PCR) tubes were mounted on a plastic holder having a 4×4 hole arrangement and this was placed exactly in the center of the coil. The sample temperature was maintained at room temperature (∼23°C). The *in vitro* MRI measurements were performed in the following order: T_1_-weighted imaging using a conventional spin echo (SE) sequence, multi-echo SE imaging for transverse relaxation time (T_2_) calculations, and an inversion-pulse prepared RARE (rapid acquisition with relaxation enhancement) sequence for longitudinal relaxation time (T_1_) calculations. **T_1_-weighted imaging**: Two sets of 2-dimensional (2D), multi-slice, T_1_-weighted images were obtained using a conventional SE sequence with the following parameters: pulse repetition time (TR)  =  400 ms, echo time (TE)  =  9.57 ms, matrix size  =  256×128 (sagittal) or 256×256 (horizontal), field of view (FOV)  =  40×20 mm^2^ (sagittal) or 40×40 mm^2^ (horizontal), slice thickness (ST)  =  1.2 mm (sagittal) or 1.0 mm (horizontal), slice gap (GAP)  =  9 mm, and number of acquisitions (NA)  =  8. Images were acquired in the sagittal (4 slices) and horizontal orientations (1 slice). The horizontal slice was adjusted so that the pelleted MNCs lay within it. The nominal voxel resolution was 156×156×1200 µm^3^ (sagittal) or 156×156×1000 µm^3^ (horizontal). **Multi-echo imaging**: 2D multi-slice multi-echo imaging was performed to generate T_2_ maps using a SE sequence with the following parameters: TR = 4000 ms, number of echoes  =  16 with TE  =  6.8, 13.6, 20.4, 27.2, 34.0, 40.8, 47.6, 54.4, 61.2, 68.0, 74.8, 81.6, 88.4, 95.2, 102.0, and 108.8 ms, matrix size  =  128×128, slice orientation  =  horizontal (same slice orientation as T_1_-weighted imaging), FOV  =  40×40 mm^2^, ST  =  1.0 mm, GAP  =  1.5 mm, and NA  =  2. The nominal voxel resolution was 313×313×1000 µm^3^ (horizontal) for these images. **Inversion recovery imaging**: Inversion pulse prepared 2D single-slice RARE imaging was performed to generate T_1_ maps with the following parameters: TR  =  16000 ms, TE  =  6.8 ms, inversion time  =  35, 50, 100, 200, 400, 800, 1600, 3200, 6400 ms, rare factor  =  4, matrix size  =  128×128, and NA  =  2. The geometry, slice orientation, and nominal voxel resolution were the same as for the horizontal multi-echo MRI.

#### *In vivo* MRI measurements

Anesthesia in rats was induced with 4.0% isoflurane and maintained with 2.0% isoflurane using a facemask. Rectal temperature was monitored using an optical fiber sensor and maintained at approximately 37.5°C with a heating pad. MRI acquisitions were performed with the same magnet and console as the *in vitro* measurements. A 75-mm-diameter birdcage transmitting/receiving coil (Bruker-Biospin) was used. The *in vivo* MRI measurements were performed in the following order: T_1_-weighted imaging using a conventional SE sequence followed by multi-echo SE imaging for T_2_ calculations and MR angiography. **T_1_-weighted imaging**: Three sets of 2D, multi-slice, T_1_-weighted images were obtained using a conventional SE sequence with the following parameters: TR  =  350 ms, TE  =  9.57 ms, matrix size  =  256×256, FOV  =  44.8×44.8 mm^2^ (the FOV was expanded up to 54.4×54.4 mm^2^ depending on animal size), ST  =  1.5 mm, GAP  =  1.5 mm, and NA  =  8. Slice orientation was axial (8 slices with 0 or 1.5 mm slice offset) and horizontal (5 slices with 0- or 1.5-mm slice offset). The nominal voxel resolution was typically 175×175×1500 µm^3^, with the lowest resolution being 212.5×212.5×1500 µm^3^. The total acquisition time for the 2 orientations was 48 min. **Multi-echo imaging**: 2D multi-slice multi-echo imaging was performed to generate T_2_ maps using a SE sequence with the following parameters: TR  =  12000 ms, number of echoes  =  8 with TE  =  10, 20, 30, 40, 50, 60, 70 and 80 ms, matrix size  =  256×256, slice orientation  =  coronal (same slice orientation as T_1_-weighted imaging), FOV  =  44.8×44.8 mm^2^ (FOV was expanded up to 54.4×54.4 mm^2^ depending on animal size), ST  =  1.5 mm, GAP  =  1.5 mm, and NA  =  2. The nominal voxel resolution was the same as for the T_1_-weighted MRI. The total acquisition time for the multi-echo imaging was 24 min. **MR angiography**: 3-dimensional gradient-echo imaging was performed to generate angiography using a spoiled gradient echo sequence (FLASH) with the following parameters: TR  =  15 ms, TE  =  2.4 ms, FA  =  20°, matrix size  =  256×128×128, FOV  =  51.2×25.6×25.6 mm^2^, and NA  =  4. The nominal voxel resolution was 200 µm in all directions. The total acquisition time for the angiography was 12.28 min.

#### Low field MRI measurements

To verify the contrast mechanism of MEMRI, acquisitions were also performed using 0.2 T MRI for an *in vitro* (n = 1) study. For the *in vitro* MNC measurements, 2D, multi-slice T_1_-weighted images were obtained using a conventional SE sequence with the following parameters: TR  =  300 ms, TE  =  25 ms, matrix size  =  256×256 (sagittal) or 256×256 (horizontal), FOV  =  200×200 mm^2^, ST  =  1.2 mm (sagittal) or 1.0 mm (horizontal), GAP  =  9 mm, and NA  =  4. Images were acquired in the sagittal (4 slices) and horizontal orientations (1 slice).

### Indium-111 labeled MNCs for SPECT imaging

To visualize the kinetics of the transplanted MNCs using SPECT, MNCs were labeled with ^111^In-oxinate (Nihon-Mediphysics, Tokyo, Japan) by incubating for 30 min at 37.0°C[39]. The radioactive half-life of the ^111^In was 2.8 days. After incubation, the cells were washed in 5% serum PBS and subjected to 400 G centrifugation for 5 min. ^111^In-oxinate labeling efficiency was then evaluated using a well counter. The cells were equally divided into 2 parts. One half was injected into the ischemic hindlimb with the other half injected into the opposite intact limb. The rat underwent *in vivo* imaging with SPECT (GCA-9300, Toshiba, Japan) 0, 2, 14, 21, and 28 days after the injection using the same anesthetic condition as for the MRI observation.

### Laser Doppler perfusion imaging

Rats were initially anesthetized with 4.0% isoflurane, after which anesthesia was maintained with 2.5% isoflurane combined with a constant air-flow at a rate of 0.5 L/min. The animals were also placed on a heating pad to maintain the body temperature. Hindlimb perfusion was measured with a laser Doppler perfusion analyzer (Moor Instruments, UK) 21 days after disappearance of Mn enhancement in the T_1_-weighted MRI (43±14 days after MNC transplantation).

### Fluorescence microscopy and immunohistochemical study

To investigate revascularisation of the tissue, the rats were euthanized with a lethal dose of pentobarbital (150 mg/kg, Dainippon-Sumitomo Pharma), after which the ischemic limbs were extracted and immediately frozen in liquid nitrogen. Typically, 20 sections were prepared in 12- µm slices for each animal and viewed with an inverted fluorescent microscope (IX71, Olympus, Tokyo, Japan). In addition, we also used rat monoclonal CD31 antibody (Serotec, Kidlington, UK) for sections prepared at days 2 and 43. All sections were carefully examined and digital photographs were obtained at 4 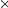, 20 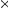, and 100 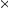 magnification at the region of highest fluorescence intensity. To assess the CD31 positive area, we analyzed a randomly selected 6 areas (0.136 mm^2^) in the transplanted site and calculated the fraction of the total area that was stained by CD31.

### Data analysis

#### MR imaging analysis

Image reconstruction and analysis were performed using ParaVision (Bruker-Biospin) and MRVision (MRVision, Winchester, MA, USA). Quantitative T_1_ maps were calculated from the inversion recovery MRI data with a non-linear least squares fitting. In addition, quantitative T_2_ maps were calculated from the multi-echo data using the same fitting. Relaxation rates (R_1_  =  1/T_1_, R_2_  =  1/T_2_) were calculated directly from the T_1_ and T_2_ maps.

The volume of the site where signal change was induced by transplanted Mn-labeled MNCs was manually calculated for every T_1_-weighted MRI slice by an independent observer using the ROI tool of the MRVision software. The observer was thoroughly educated about the anatomical features of the injection site. Although the threshold for selecting the boundary of the affected area was generally set at 2 standard deviations of the peripheral muscle signal, the observer manually determined the profile-line when the images were complicated by both positive and negative signal enhancement. MR angiography was visualized using a maximum intensity projection (MIP) that was calculated from the 3D-MRI data with the ParaVision tool. With reference to the 3D-MIP data, the number of arteries was manually counted in each of the eight transverse slices of the 2D angiography data covering the distal hindlimb. The mean number of arteries was then calculated.

#### Statistics

All results are expressed as the mean ± standard error of the mean. Statistical comparison was performed using two-way analysis of variance (ANOVA) with the Bonferroni post-hoc test. The statistical calculations were performed using commercial analysis software (GraphPad Prism, GraphPad Software, San Diego, CA, USA). Statistical significance was set to P<0.05.

## Results

### Relaxation times and contrast of MnCl_2_-labeled MNCs

**Figure 1:**
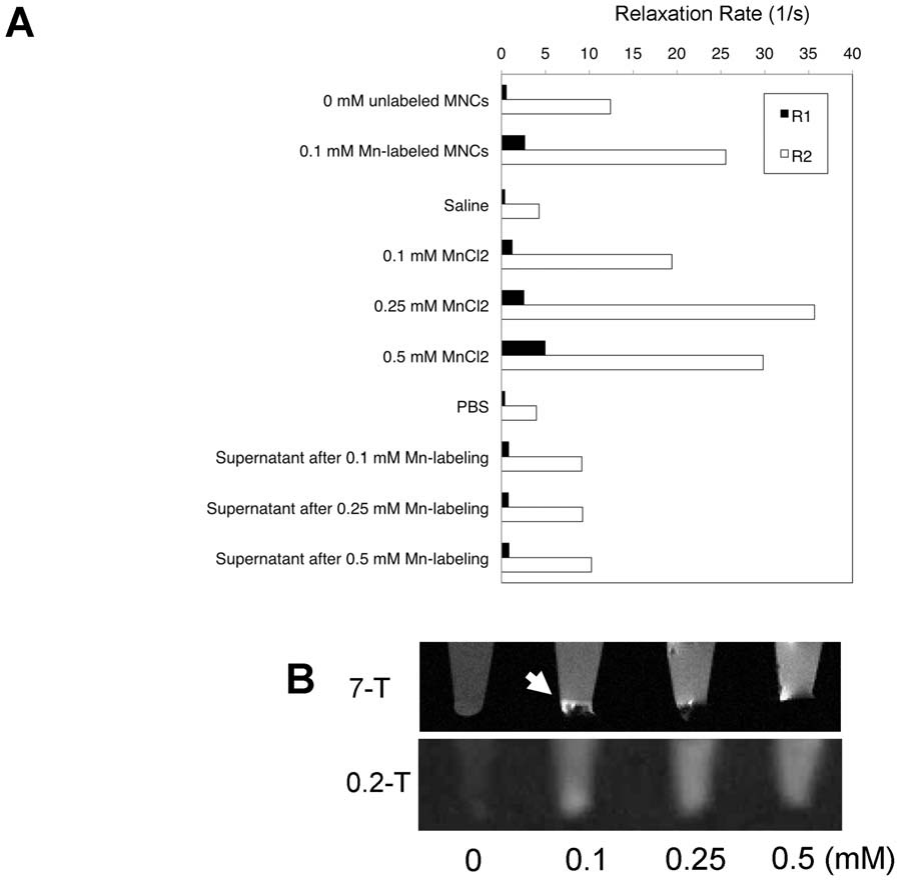
Relaxation times and contrast of Mn-labeled MNCs. (A) R_1_ and R_2_ calculated for the *in vitro* samples are presented. The pelleted 0.1 mM Mn-labeled MNCs showed larger R_1_ and R_2_ in comparison to the saline control. It was not possible to calculate the R_1_ and R_2_ values for the MNCs with 0.25 mM Mn-labeling and over (N/A) because the T_2_ was too short due to the large susceptibility effect. The R_1_s of the MnCl_2_ solutions were approximately linearly correlated with the Mn concentration. The R_1_ and R_2_ for the supernatant fluid were approximately independent of the initial Mn labeling concentration. (B) T_1_-weighted sagittal images of pelleted MNCs after suspension in 0, 0.1, 0.25, and 0.5 mM Mn solutions. The pelleted MNCs at 0.1 mM MnCl_2_-labeling showed partial signal enhancement in the T_1_-weighted 7 T MRI (arrow). The pelleted MNCs with MnCl_2_-labeling over 0.25 mM lost signal in comparison with the unlabeled (0 mM) control due to the very short T_2_ and the T_2_* susceptibility effect at 7 T. In the 0.2 T MRI, the the 0.1, 0.25 and 0.5 mM MnCl_2_-labeled MNCs showed positive contrast, although there may have been some signal loss at the base of the 0.5 mM sample.

*In vitro* relaxation rates for the pelleted Mn-labeled MNCs, MnCl_2_ solution, and supernatant MnCl_2_ fluid after labeling are shown in Fig. 1A. An increase of both R_1_ and R_2_ was observed for the pelleted 0.1 mM Mn-labeled MNCs in comparison with the un-labeled MNCs (Fig. 1A). It was not possible to calculate the R_1_ and R_2_ values for the MNCs with 0.25 mM Mn-labeling and over because the T_2_ was too short due to the large susceptibility effect. The R_1_s of the MnCl_2_ solutions were approximately linearly correlated with the concentration (Fig. 1A). The supernatant fluid remaining after Mn-labeling of MNCs was also measured to estimate the amount of Mn discharged from the MNCs. Both R_1_ and R_2_ for the supernatant fluid were approximately independent of the initial Mn labeling concentration (Fig. 1A). The results and trypan-blue staining (viability 95.4±2.4%, 0.25 mM MnCl_2_-labeled MNCs) suggest that there was limited Mn release from labeled cells and little cytolysis for 0.1–0.5 mM MnCl_2_-labeling.

Typical T_1_-weighted images of pelleted MNCs are presented in Fig. 1B. In the 7-T T_1_-weighted MRI, the 0.1 mM MnCl_2_-labeled MNCs showed an upper layer of positive signal enhancement. The 0.25 and 0.5 mM MnCl_2_-labeled MNCs suffered signal loss in comparison to the unlabeled (0 mM MnCl_2_) controls. On the other hand, in the 0.2 T T_1_-weighted image the 0.1 and 0.25 mM MnCl_2_-labeled MNCs showed uniform positive signal enhancement, while there may have been some signal loss at the base of the 0.5 mM sample. These results indicate that the signal loss was not caused by Mn discharge from the cells or cytolysis, but by the T_2_ shortening and T_2_* susceptibility effects caused by the labeled MNCs. In preparation for the *in vivo* experiments, we performed an *in vivo* trial with both 0.125 and 0.25 mM MnCl_2_-labeled MNCs. As the enhancement due to the 0.125 mM MNCs disappeared within 1 day of transplantation (data not shown), a concentration of 0.25 mM MnCl_2_ was used to label MNCs for the *in vivo* experiments.

### Short-term *in vivo* tracking of Mn-labeled MNCs in the ischemic hindlimb

**Figure 2:**
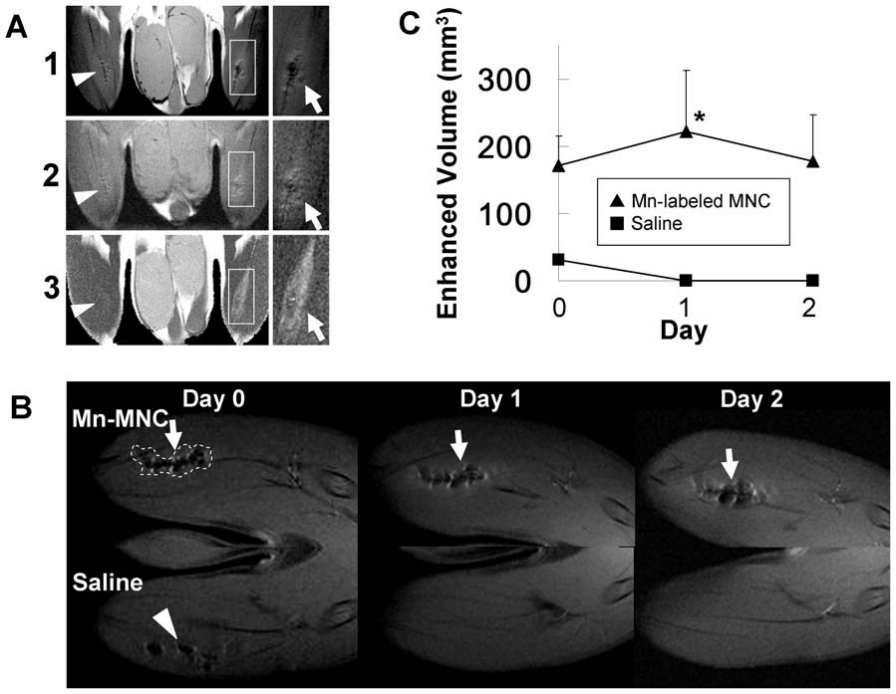
Typical MR images immediately after transplantation of Mn-labeled MNCs and short-term changes of the signal intensity in ischemic rat legs. A typical horizontal T_1_-weighted image (A1), proton density image (A2), and calculated T_2_ map (A3) are presented for the same animal immediately after the injection of Mn-labeled MNCs. The images on the right are magnifications of the area inside the white rectangles. Mn-labeled MNCs (white arrows) were injected into the left leg (right side of the images) and saline (white arrow heads) was injected into the right leg (left side of the images). (B) Short-term observation of T_1_-weighted signal enhancement in ischemic rat legs is presented. The upper figures show horizontal T_1_-weighted MRI after Mn-labeled MNC injection from 0 to 2 days after the transplantation. The lower figures show T_1_-weighted MRI after saline injection in the same animal (contralateral leg). The area enclosed by the white dotted lines is an example of a typical region of interest (ROI). At both 1 and 2 days after injection, Mn-labeled MNCs were visualized as a “double-layered structure” that has a positive outline and dark core (B, white arrows). The signal reduction after saline injection soon disappeared (B, white arrow head). (C) Volume change after the MNC injection. The filled triangles indicate volume change at the Mn-labeled MNC injected site and the filled squares correspond to the saline injected site. * Significant difference vs. volume of saline injected control, level of significance P<0.05 for two-way ANOVA with Bonferroni post-hoc test (n = 3).

Figure 2A presents typical MR images immediately after transplantation of Mn-labeled MNCs. Signal loss was observed at the transplanted site in T_1_-weighted (A1) and proton-density (A2) images. Signal enhancement surrounding the ‘dark’ transplanted site was also observed in the same images. Furthermore, shorter T_2_ at the MNC injected site and longer T_2_ at the MNC injected muscle were observed (A3). Figure 2B and 2C present the dynamics of Mn-labeled MNCs in comparison with saline (as a control) from immediately after intramuscular administration to 2 days later. Immediately after the injection, both Mn-labeled cells and saline provided signal reduction on the T_1_-weighted MRI (Fig. 2B). At 24–48 h after the injection, Mn-labeled cells were clearly detected as a “double-layered structure” that has a dark core with surrounding positive enhancement. The volume of the transplanted MNCs was unchanged 1 day after the transplantation, but the volume of the injected saline sites shrank to zero within 1 day (Fig. 2C).

### Long-term *in vivo* tracking of Mn-labeled MNCs in the ischemic hindlimb

**Figure 3:**
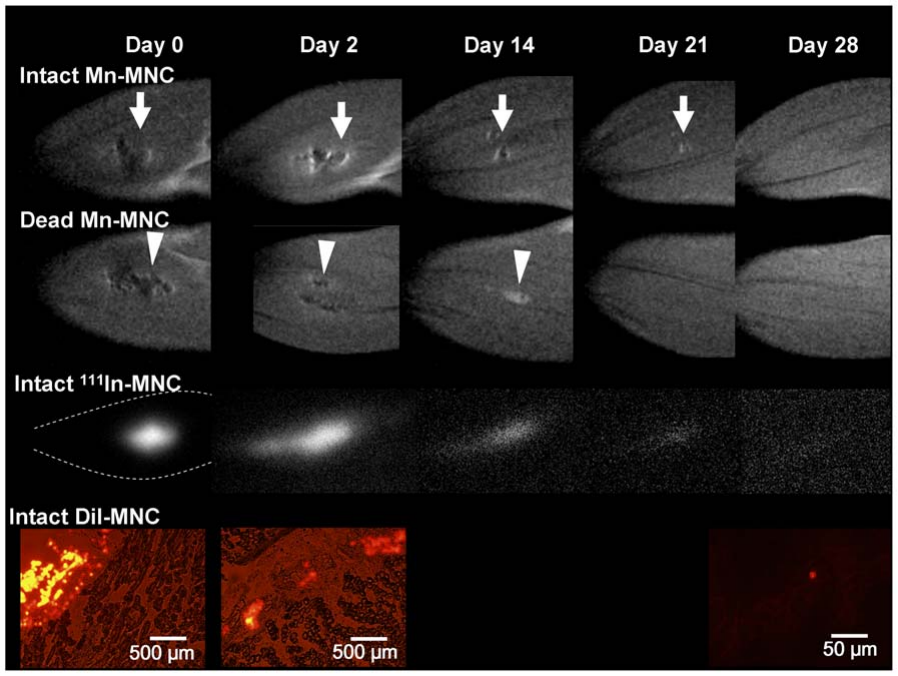
Long-term *in vivo* observations after transplantation of Mn-labeled MNCs in ischemic rat legs. T_1_-weighted images of intact (1^st^ row) and dead (2^nd^ row) Mn-labeled MNCs, SPECT images of intact ^111^In oxine labeled MNCs (3^rd^ row), and fluorescence microscopy images of intact DiI-labeled MNCs (4^th^ row) are presented. The images were obtained between 0 and 28 days after administration. The intact Mn-labeled MNCs were typically observed for at least 7 and up to 21 days in the injected muscle (arrows). The dead Mn-labeled MNCs (arrow heads) tended to decrease in volume earlier than the intact MNCs.

**Figure 4:**
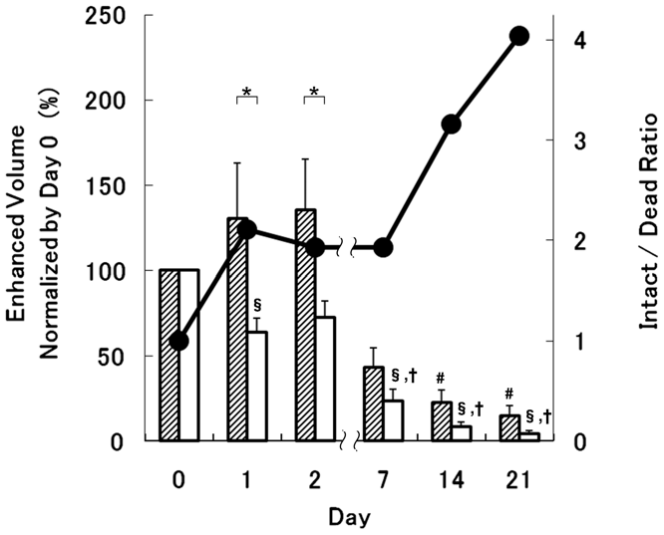
Comparison of the enhanced volume in T_1_-weighted MRI due to intact and dead Mn-labeled MNCs. The shaded and white bars indicate the enhanced volume (normalized by the volume measured at day 0) of the intact and dead Mn-labeled MNCs, respectively. The enhanced volume of the intact MNCs shrank slower than that for the dead MNCs (bar-graph). The ratio of intact-to-dead MNCs is also superimposed as a line-graph (black circles, right vertical axis). An intact/dead ratio of “1” means that the enhanced volumes on the T_1_-weighted MRI are the same for the Mn-labeled intact and dead MNCs. The intact/dead ratio tended to increase with time. * Significant difference (P<0.05) between the enhanced volumes of the intact and dead MNCs, two-way ANOVA with Bonferroni post-hoc test (n = 6). # Significant difference (P<0.05) vs. intact MNCs (day 1) § Significant difference (P<0.05) vs. dead MNCs (day 0) † Significant difference (P<0.05) vs. dead MNCs (day 1)

Figure 3 demonstrates the long-term dynamics of transplanted MNCs using MRI for both intact and dead Mn-labeled cells, SPECT for intact ^111^In-labeled cells, and fluorescence microscopy for DiI-labeled cells from 0 to 28 days after the intramuscular administration. The viability of the MnCl_2_-labeled intact MNCs before transplantation was more than 95.4±2.4% in all experiments (trypan-blue staining). The intact Mn-labeled MNCs were observable for at least 7 and up to 21 days in the injected muscle. This result agreed with the changes to the radioactive signal measured by SPECT using ^111^In-labeled MNCs. The enhanced volume (normalized by the volume at day 0) of intact Mn-labeled MNCs was significantly larger than the enhanced volume of dead Mn-labeled MNCs for the first 1–2 days, and then both gradually shrank (Fig. 4, bar graph), although the enhanced volume of the dead Mn-labeled MNCs tended to shrink earlier than the intact MNCs. The ratio of intact-to-dead MNC enhanced volume gradually increased until 21 days after the transplantation (Fig. 4, line graph). Fluorescent DiI-labeled MNCs were observable for 0–2 days after the injection (Fig. 3). Although careful experiments were performed at 14 or 21 days, the fluorescence from DiI was not detected at those times. A very small volume of DiI-labeled MNCs was observed at 28 days after the injection.

### Chronic evaluation of blood flow recovery after MNC transplantation

**Figure 5:**
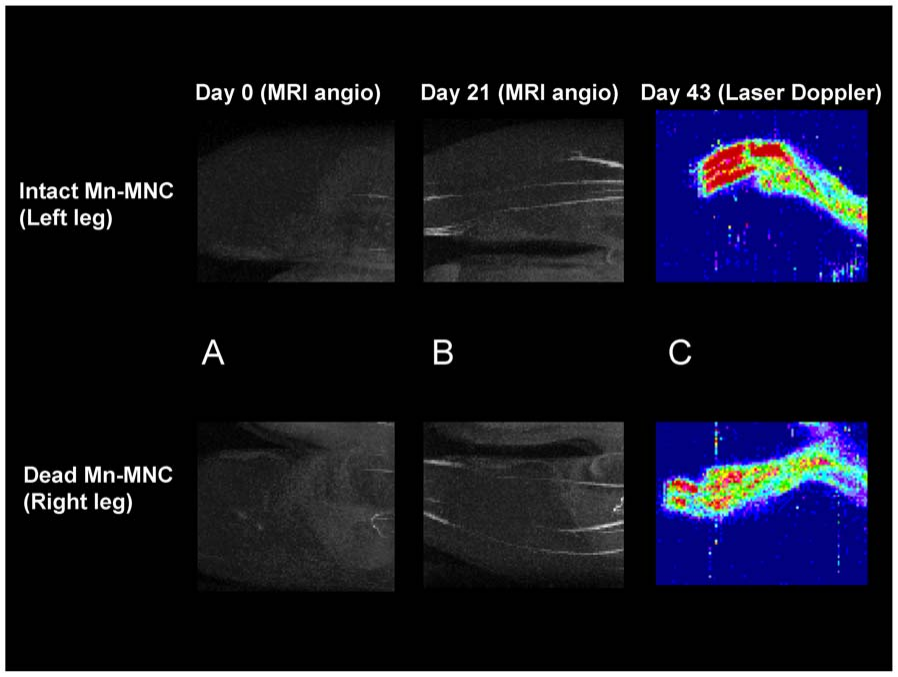
Chronic evaluation of angiogenesis using MRI angiography and Laser Doppler perfusion imaging. Representative MRI angiographs (A and B) and laser Doppler perfusion images (C) are presented. The MRI angiographic images were obtained at 0 (A) and 21 (B) days after muscle ischemia and MNC transplantation. Recovery of arterial blood flow was observed in the hindpaw for both the intact and dead MNC administered legs at 21 days. The intact MNC transplanted side showed a similar number of arteries to the contralateral side containing dead MNCs. The laser-Doppler perfusion image was acquired 43 days after the ischemia and transplantation (C). The muscle perfusion measured with laser-Doppler in the left legs (injected with intact MNCs) was 20±9% (n = 6) higher than that in the right legs (dead MNCs).

**Figure 6:**
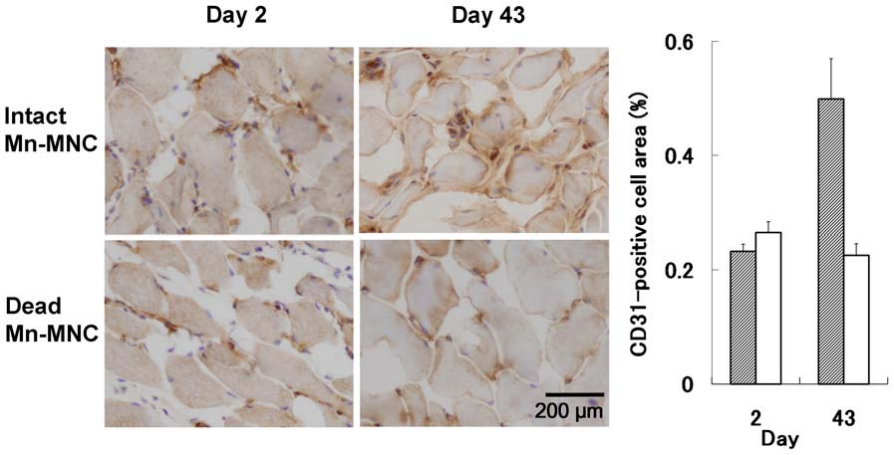
Immunohistochemistry for CD31. Both intact and dead Mn-MNC implanted sites were stained with CD31 at 2 and 43 days. Necrotic myofibers and neutrophilic infiltration induced by hindlimb ischemia were observed in both the intact and dead Mn-MNCs implanted sites at day 2. The bar graph indicates the fraction of the total area covered by CD31-positive cells (brown) of the intact (shaded) and dead (white) Mn-labeled transplanted MNCs. The areas coevered by CD31-positive cells in the intact Mn-MNCs implanted site at 43 days was larger than that at 2 days or in the dead Mn-MNCs implanted site at 43 days.

Figure 5A and B show typical angiography in the affected legs. Recovery of arterial blood flow was observed for both the intact and dead MNC administered legs at 21 days (Table 1). There was no significant difference in the number of arteries between the sites where intact and dead MNCs were transplanted (Fig. 5A and B, Table 1). The ratio of muscle perfusion in the left and right legs (intact-to-dead MNCs) measured by laser Doppler 43 days after the transplantation was 120±9% (n  =  6) (Fig. 5C). After immunohistochemical staining for CD31 (as a marker of capillary endothelial cells as well as neutrophil and macrophages), the area covered by CD31-positive cells (brown) in the intact Mn-MNCs implanted site at 43 days was larger than that at 2 days or in the dead Mn-MNCs implanted site at 43 days (Fig. 6).

**Table 1:** Evaluation of MR angiography after transplantation of manganese-labeled mononuclear cells.

	Control(dead)(n = 6)	Therapy(intact)(n = 6)	P
Number of arteries (Day 0)	6.10±0.63	6.40±0.87	0.664
Number of arteries (Day 21)	8.56±0.91*	9.19±0.95*	0.172

Values are mean ± SE.

Significant difference vs. Day 1 group, level of significance P<0.05

(*) for paired T test.

## Discussion

This is the first report describing the transplantation of Mn-labeled MNCs to a level sufficient for detection over a period of several weeks *in vivo* by MRI. There were 2 major findings in this study: 1) Mn-labeled MNCs were continuously observed by MRI for at least 7 and up to 21 days after transplantation in the ischemic leg. This was similar to the ^111^In-labeled MNCs visualized by SPECT. 2) The volume of the site injected with dead MNCs was significantly smaller than that injected with intact Mn-labeled MNCs at 1–2 days after the transplantation, although the dead Mn-labeled MNCs were also found for approximately 2 weeks in the ischemic legs.

Mn has been shown to be an effective cell-labeling agent for lymphocytes[36], isolated pancreatic beta-cells[40], and hepatocytes using Mn(III)-transferrin[41] *in vitro* and luciferase-transfected human embryonic stem cells *in vivo* [42]. Although cell-tracking using quantitative radioisotope imaging has been attempted[43], it is difficult to observe signal changes non-invasively over a period of weeks due to the limited radioactive half-life. In addition, the spatial resolution of clinical SPECT is not as good as that of MRI. Therefore, MRI provides a more precise evaluation for human angiogenic therapy using cell transplantation.

Clinical trials of MNC transplantation therapies have been reported and some technical improvements noted[9], [10]. In order to develop more effective cell therapies, the location, distribution and function of these cells must be determined in a non-invasive manner. MRI tracking of magnetically labeled cells following transplantation or transfusion may fulfill this requirement[14], [19], [20]. In the present study, it was demonstrated in a rat model of hindlimb ischemia that Mn-labeled MNCs are useful for *in vivo* tracking of the implanted cells with possible therapeutic benefits to perfusion recovery due to neovascularization (Figs. 5, 6).

The transplanted Mn-labeled MNCs were typically detected as a “double-layered structure” having a negative core with surrounding positive enhancement in the T_1_-weighted and proton-density MRI immediately after the intramuscular administration (7-T MRI, Fig. 2A1, A2). Previous trials have indicated that Mn^2+^ at a concentration of 1.0 mM in T-lymphocytes can provide positive contrast in T_1_-weighted MRI at 11.7 T[36]. On the other hand, Mn-labeled B lymphoblastoid cell-lines provided signal loss in 1.0–2.0 mM MnCl_2_ suspensions[36]. There are several differences, such as cell type and cell density between previous and the present *in vitro* trials of MnCl_2_ labeling. Some heme iron from the red blood cells may have been retained with the MNCs. In this study, a concentration of 0.25 mM MnCl_2_ was selected for the legs so as to allow long-term visualization *in vivo*. Even though this concentration may produce a dark core, the location of the transplanted MNCs is easily recognized. Signal enhancement (corresponding to longer T_2_) was observed in the MNC injected muscle on T_2_-werighted MRI (Fig. 2A3). The enhancement indicates that MNC transplantation induced edema and/or gradual inflammatory responses in the muscle.

The dark core of the “double-layered structure” turned positive 2–3 weeks after transplantation in the leg (Fig. 3). Because the same structure was also present for the saline treated group in the acute stage, it is speculated that the dark core evident at 24 h after administration was predominantly caused by T_2_* shortening[17] due to tissue damage from the injection or bleeding (Fig. 2B). In the chronic stage, the mechanism responsible for the dark zone is not clear, although migration of endogenic phagocytes and subsequent englobement may be involved. The positive outline extended and became increasingly blurred over the first 1–2 days, after which the “double-layered structure” typically became a simple positive enhancement in the T_1_-weighted MRI for up to 21 days (Fig. 3). This result demonstrates that intramuscularly-administered Mn-labeled MNCs contribute to MRI contrast as positive (T_1_-weighted) signal enhancement, with the dark core (sometimes accompanied by inhomogeneous signal enhancement) at the injected site. It is expected that imaging at lower fields, such as those typically found in clinical scanners, will provide mainly positive signal enhancement because the T_2_* susceptibility effect is not as strong.

The kinetics and volume change of the intact Mn-labeled MNCs were similar to that of the ^111^In-labeled MNCs measured by SPECT. The experiments with ^111^In-oxine, which never leaks from labeled cells, support the hypothesis that signal change in the T_1_-weighted MRI is predominantly caused by the distribution and density of labeled MNCs. The kinetics and presence of each cell was also verified under a microscope with the fluorescence DiI-labeled MNCs at 0 and 2 days after administration. Although no fluorescence signal could be found at 7–21 days, it is speculated that the number of DiI-labeled MNCs gradually decreased with time because only a small amount of fluorescent signal was observed at 28 days after transplantation.

There are some limitations to the accuracy of tracking Mn-labeled MNCs. First, it was difficult to evaluate the injected volume exactly for the first day. This was because the saline treated group also had a small contrast change, meaning that the injected solvent, small tissue damage and/or bleeding also contributed to the predominantly dark contrast. This becomes more important with high field MRI because of large susceptibility differences. Nevertheless, since the contrast generated by the saline disappeared at 1 day after the injection, the double-layered signal enhancement must be caused by the Mn-labeled MNCs beyond that time (Fig. 2B and 2C).

Second, Mn^2+^ may be gradually discharged from labeled MNCs and transported to other cells such as myocytes and macrophages. The *in vivo* kinetics of Mn^2+^ may mimic that of Ca^2+^ because Mn^2+^ has an ionic radius similar to that of Ca^2+^ and is handled similarly in many biological systems[25]. Mn^2+^ can accumulate via Ca^2+^ channels and be stored in the mitochondria for long periods[25]. It is also known that the rate of discharge from neuronal cells is very slow[29]. Therefore, even though Mn^2+^ may leak from the MNCs, the discharge rate is probably limited.

Third, it has been reported that the survival rate of grafted skeletal-muscle precursor cells steadily decreased to 14.6±1.1% in 24 h and to 7.9±0.6% in 72 h[44]. In addition, *in vivo* optical bioluminescence imaging has shown the degradation of Mn-labeled cell viability after transplantation[42]. In other words, although 95.4±2.4% of the MnCl_2_-labeled MNCs (0.25 mM) were intact before transplantation, most of the implanted cells died within 24 h and were engulfed by phagocytes. This means that the enhanced volumes of both intact and dead MNCs after transplantation include unknown numbers of dead cells (Fig. 4, bar graph). The present results showed that, although the area displaying contrast due to the dead MNCs shrank faster than that of the intact MNCs, the dead Mn-labeled MNCs could still be observed for 2 weeks (Figs. 3, 4). This non-distinctive result may be caused by the low survival rate of cells after transplantation. The different periods of enhancement for the intact and dead cells (Fig. 3) suggests that time-course analysis of MEMRI can detect small differences in the survival ratio (8∼15%) of transplanted cells.

Despite these limitations, cell labeling using Mn contrast agents could be a useful tool for tracing transplanted cells *in vivo*. Further improvement in the Mn-labeling method, such as higher survival rate of transplanted cells and a method to stabilize Mn inside cells, will allow tracking of cells and viability for evaluation of the therapeutic effects. Iron oxide nanoparticles have also been used to track injected cells for long periods after transplantation, even when only a single cell is labeled[45]. The iron-oxide-labeled cells remain observable for longer than those labeled with Mn[19], [20]. On the other hand, Mn^2+^ has been proposed as a cell viability indicator for cardiac applications because Mn only accumulates in living cells[35], [46]. In addition, (depending on the concentration) Mn^2+^ mainly positively enhances the exact site of the transplanted cells without image distortion due to susceptibility differences in T_1_-weighted MRI. Also, although negative contrast was occasionally observed at 7 T, our results at 0.2 T suggest that mainly positive enhancement will be detected in the low magnetic fields used for clinical MRI. This property can be used to detect and distinguish the target in heterogeneous tissues and hollow organs (e.g., alimentary canal) because there are many dark regions such as air spaces, vessels and bone. Moreover, the Mn-labeling method is very simple and can be applied to any cell strain, including non-phagocytic cells, because it only requires suspension in a MnCl_2_ solution for 15–60 min.

Many papers have reported the toxicity of Mn in cells[24], [47]. As observed by inotropic status, Mn toxicity is expected for concentrations of more than 1 mM in the myocardium[33], [42]. Mn toxicity has also been investigated in human lymphocytes[36] and luciferase-transfected human embryonic stem cells[42]. No cytotoxic effect was observed for cells suspended for 60 min in MnCl_2_ concentrations under 0.5 mM[36]. Therefore, suspension of cells in 0.25 mM MnCl_2_ for 60 min can produce sufficient Mn-labeling while also avoiding possible cell toxicity. In the present report, counting cells stained with trypan-blue revealed a viability of intact MNCs of 95.4±2.4%. However, the double-layered structure created by the labeled MNCs (Fig. 1B) suggests that intracellular Mn concentrations (such as intra-mitochondrial Mn) may be greater than 0.5–1.0 mM because MNCs have phagocytic activity. Further investigation of intracellular Mn concentration is required to allow safer and effective Mn-labeling experiments.

The present study used 0.28 mg of MnCl_2_ for the labeling procedure. This suggests a method for Mn-labeling for clinical applications because digestion of 2.3 mg/d of Mn is recommended for adult humans[48], and the ‘tolerable daily intake’ of Mn is 4.38 mg/d[49]. Further studies of cell labeling with small doses of Mn are warranted to prepare for clinical trials.

In conclusion, intramuscular transplanted Mn-labeled MNCs were observed in both of the ischemic legs of rats for over 3 weeks using 7-T MRI. Observation of Mn-labeled MNCs was verified by comparing the MRI findings with SPECT and fluorescence microscopy. The dynamics of intact Mn-labeled MNCs agreed well with the SPECT imaging of ^111^In-labeled MNCs in the ischemic legs. The present Mn-enhanced method enabled visualization of the transplanted area with a 150–175 µm in-plane spatial resolution and allowed the migration of labeled-MNCs to be observed for long periods in the same animal. Although it still needs to be investigated whether the Mn-labeling technique can reflect the cell viability, after further optimization MRI-based Mn-enhanced cell-tracking could be a useful technique for evaluation of cell therapy both in research and clinical applications.
